# Concerns of Quality and Reliability of Educational Videos Focused on Frailty Syndrome on YouTube Platform

**DOI:** 10.3390/geriatrics7010003

**Published:** 2021-12-23

**Authors:** Natalia Maria Hawryluk, Małgorzata Stompór, Ewelina Zofia Joniec

**Affiliations:** Department of Family Medicine and Infectious Diseases, Medical Faculty, Collegium Medicum, University of Warmia and Mazury, 10-719 Olsztyn, Poland; mstompor@tlen.pl (M.S.); ewelina.joniec@gmail.com (E.Z.J.)

**Keywords:** frailty syndrome, YouTube, internet, patient education, social media

## Abstract

(1) Background: Evaluation of the quality and reliability of the frailty syndrome videos available on YouTube platform was the aim of this study. (2) Methods: The observational study included 75 videos retrieved by searching seven terms related to frailty syndrome on YouTube. The quality and reliability of the videos were measured using three different tools: quality criteria for consumer health information (DISCERN), the Global Quality Score (GQS), and the Journal of American Medical Association (JAMA). The video content was categorized according to the following characteristics: video provider, duration, view count, average daily views of the video, average daily views of a channel, channel subscribers, number of days since upload date, likes, dislikes, comments, the external webpages linked to the videos. (3) Results: The videos had a mean duration of 375 s and an average number of views of 1114. The quality of 17 videos assessed in the study was found to be high, 48—intermediate, and 10—low. The high-quality videos had the longest duration, the highest number of views, and points for the DISCERN score. The physician uploaders had the highest mean DISCERN and mean GQS scores, the highest number of views, and the longest duration but the hospital channels had the highest JAMA score. (4) Conclusions: YouTube can be a valuable source of medical information for patients and caregivers. The quality of videos mostly depends on the authorship and the source of video providers—physicians, academic, and health care-related organizations provide the best quality content based on professional medical knowledge.

## 1. Introduction

Frailty is an important geriatric syndrome that affects about 18% of the European population aged 65 and over [1]. It was defined by the World Health Organization as a “state in which the ability of older people to cope with every day or acute stressors is compromised by an increased vulnerability brought by age-associated declines in physiological reserve and function across multiple organ systems” [2]. The main symptoms of frailty are unintentional weight loss, decrease in muscle mass, deterioration of nutritional status, weakness, slowness, fatigue, low level of physical activity, and cognitive impairment [3,4,5]. Frailty syndrome is associated with an increased risk of falls, disability, hospitalization, post-surgical complications, and mortality [6,7]. A healthy lifestyle, physical activity, and balanced diet avoiding smoking and excess alcohol intake help to reduce the risk of frailty [4].

About 45% of adults search the Internet for health information [8]. Recently, papers have been published indicating YouTube, the online video-sharing and social media platform, as a valuable source of information about many symptoms and diseases (including sarcopenia, narcolepsy, disc herniation, breast self-examination, and many others) [9,10,11,12]. Several online materials concerning frailty syndrome can also be found on this platform, but their quality and reliability have not been formally assessed yet. Evaluation of the quality and reliability of the frailty syndrome videos available on the YouTube platform with the use of widely approved tools such as DISCERN, the Global Quality Scale, and JAMA benchmark criteria combined with content-focused questions was the aim of this study.

## 2. Materials and Methods

**Figure 1:**
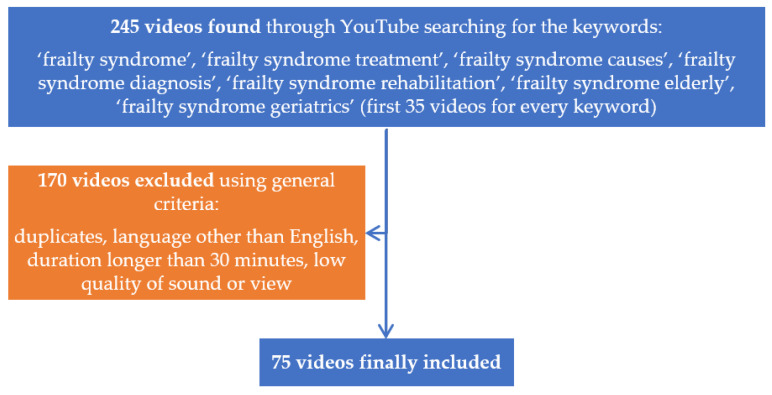
Flowchart shows selection of YouTube videos for the study.

YouTube (www.youtube.com) was searched on 15 April 2021 for the keywords: ‘frailty syndrome’, ‘frailty syndrome treatment’, ‘frailty syndrome causes’, ‘frailty syndrome diagnosis’, ‘frailty syndrome rehabilitation’, ‘frailty syndrome elderly’, ‘frailty syndrome geriatrics’ to identify related video materials. The search results were the first 35 videos for every keyword. Links to 245 videos were copied and transferred to a Microsoft Excel spreadsheet. Videos that were non-English-language, longer than 30 min, with low quality of sound or picture, and multiple repetitions were excluded from the assessment. Finally, 75 videos were included in the study (Figure 1). The videos were evaluated independently by two authors (NH, EJ). The raters were blinded to each other’s evaluations, so consultation was not possible. Before the beginning of the study, the raters studied and worked with the evaluation tools. We analyzed each video according to the following characteristics: video provider, content and duration of the video, view count, average daily views, average daily views of a channel, channel subscribers, the number of days since the upload date, likes, dislikes, comments, the external webpages linked to the videos [10]. The content of the videos included: frailty syndrome definitions, risk factors, pathophysiology, scales to diagnose the problem, epidemiology, prevention (including diet programs and exercise). Video providers were classified as physicians, hospitals, health, educational, and news channels. Additionally, all videos were divided into two groups: physician as a speaker and without physicians. To evaluate the popularity of the videos, we used the Video Power Index (VPI). Average daily views, which is also called the view ratio, were calculated using the following formula: view count/days since upload, and the like ratio was determined using the formula: like × 100/(like + dislike). The VPI values were also calculated according to the formula: VPI = like ratio × view ratio/100.

To evaluate the basic quality and reliability of the videos, three different tools were used: quality criteria for consumer health information (DISCERN), the Global Quality Score (GQS), and the Journal of American Medical Association (JAMA) benchmark criteria.

The GQS scale is used to rate the quality of website resources. The GQS adopts a scoring system: 1 = poor quality, poor flow, most information is missing, and not helpful for patients; 2 = generally poor, some information is given but of limited use to patients; 3 = moderate quality, some vital information is adequately discussed; 4 = good quality, good flow, the most relevant information is covered, useful for patients; 5 = excellent quality and flow, beneficial for patients. The videos rated with 4 to 5 points were considered high quality videos, 2.5 to 3.5 points as intermediate quality videos, and those with 1 to 2 points as low-quality videos.

The DISCERN scale was used to determine reliability. DISCERN is a scoring system consisting of 16 questions with a score of 1 to 5 points per question (Table 1) [13].

Both quality and reliability were assessed as well according to the JAMA criteria, which consist of 4 items: ‘authorship, attribution, disclosure, currency’. By assigning 1 point for the fulfillment of each criterion, the total JAMA benchmark score was calculated (Table 2).

### Statistical Analysis

The licensed TIBCO Statistica software (Version 14.0.0.15, TIBCO Software Inc., Cracow, Poland) was used for statistical analysis of the results. The variables were expressed as mean ± standard deviation, numbers, and percentages. The comparison between the respective parameters of videos uploaded by physicians and others was made using the Mann–Whitney U test. Intraclass correlation coefficient was carried out to ensure inter-rater agreement of the two independent researchers. The significance level was adopted as *p* < 0.05.

## 3. Results

Among the 245 videos screened, 75 videos met the inclusion criteria. The total number of views of all the videos was 83,573, and the average number of views equaled 1114 (SD = 1876). The total duration of the videos was 28,148 s, with a mean duration of 375 s (SD = 347). The average time since upload was 1285 days (SD = 893). The total number of likes equaled 456, with an average of 6 (SD = 10). There were 16 dislikes and 31 comments. The mean like ratio was 0.7 (SD = 0.4), and the mean VPI was 1.1 (SD = 2.9).

According to the GQS, 17 videos were scored as high-quality videos (23%), 48—intermediate quality (64%), and 10—low quality videos (13%) (Table 3). The high-quality videos had the longest duration, the highest number of views and points on the DISCERN scale (Table 4). The links to the high-quality videos are given in Table A1.

**Figure 2:**
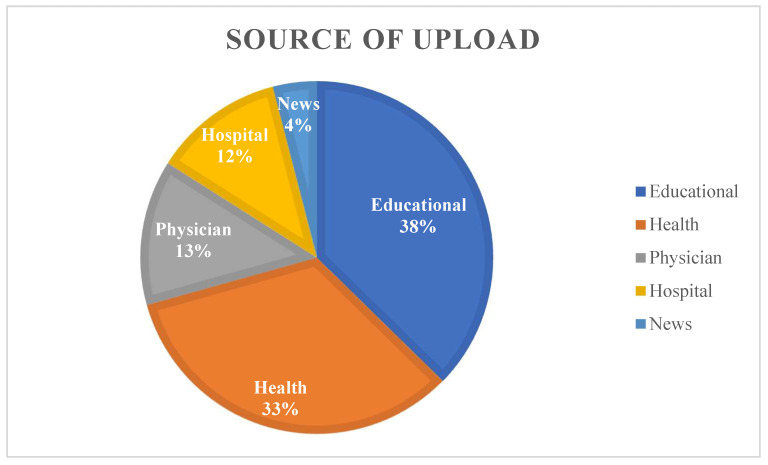
The diagram of sources of uploading.

The video uploaders consist of physicians, hospitals, health, educational, and news channels. The majority of videos were uploaded on educational channels (n = 28, 38%) (Figure 2). The physicians’ channels had the highest ratio as the source of upload in the high-quality group (50%) (Table 3). Moreover, the physician uploaders had the highest mean DISCERN and mean GQS scores, the highest number of views, and the longest duration but the hospital channels had the highest JAMA score (Table 5).

Additionally, the division of videos into two groups as physician speaking videos (n = 49, 65%) and without physicians (n = 26, 35%) shows that the average GQS score of the videos with physician speaking was 3.3, the average DISCERN score was 3.5 and JAMA score was 2.9. The average GQS score of the videos without physicians was 2.35, the average DISCERN score—3.16, and JAMA score—2.3. Statistically significant differences were found in terms of the GQS, DISCERN, and JAMA scores of videos with physician speaking compared to videos without physicians (*p* < 0.005).

**Figure 3:**
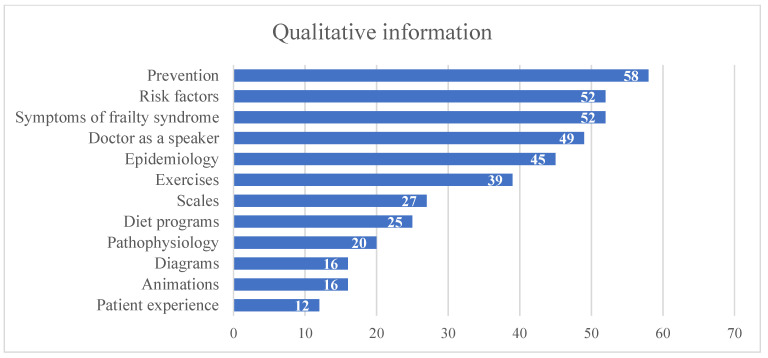
The summary of video content.

The summary of video contents is shown in Figure 3. According to the possible management of the problem of frailty, the prevention of frailty syndrome was mentioned only in 58 videos (77%), and in 12 videos (12%), the patient experience was shown. Sixteen videos (21%) contained animations and, in 27 videos (36%), diagnostic scales were used.

## 4. Discussion

Frailty syndrome is a common clinical problem, particularly in old age. The awareness and knowledge of frailty among patients, their caregivers, and medical staff should be higher. To meet this problem, patients and caregivers may try to use the YouTube platform as a valuable source of information. In 2020, there were over 2 billion YouTube users all over the world and these statistics keep growing [14]. The advantages of this platform include free and easy access, but on the other hand, the information published may not always be reliable and trustworthy.

In the presented study, we aimed to assess the quality of YouTube videos about frailty syndrome. According to the GQS, the quality of videos was mostly intermediate (64%), but in many previously conducted studies, for example, related to narcolepsy, disc herniation, and breast self-examination, researchers stated that the quality of the videos was predominantly low [10,11,12]. Out of 75 videos in the presented study, 17 were classified as a high-quality video and 10 as low-quality ones.

The videos scored as having the highest quality were uploaded by physicians or, at least, physicians contributed to them as speakers. Among those videos, the quality was intermediate or high; none of them were considered low-quality. Cetin et al. in their study analyzing videos on hypoglycemia management also reported that videos uploaded by physicians were high in quality [15].

Akyol and Karahan in their study on sarcopenia videos, also stated that the movies uploaded by physicians and academic organizations were considered high-quality [9]. In an analysis of videos dedicated to narcolepsy, Szmuda et al. reported that most of the videos were published by educational channels similar to our study [10]. Over half of the videos raised issues of prevention, risk factors and symptoms of frailty syndrome. Onder et al. assessed videos about gout and reported that the majority of them were high-quality and provided useful information for patients [16]. The same was observed in an assessment of videos about systemic lupus erythematosus published by Hsuen and colleagues: 67.8% of them were useful and only 11.5% were misleading. Similar to our study, in Hsuen’s survey, the duration of high-quality videos was longer, and the number of views was higher compared to low-quality videos [17]. On the other hand, other researchers found that 62% of videos focused on breast self-examination were misleading and the majority of them were uploaded by individual users [12]. A study by Erdem et al. who analyzed videos about kyphosis found that academic videos had higher quality, but their popularity (VPI) was lower; however, we found that the higher the quality of the videos was, the more views they had [18]. In our study, 16 videos (21%) contained animation; this percentage was higher (32%) in the paper of Gokcen et al. analyzing the quality of information on disc herniation [11].

There were limitations of this study. First, only English-language videos were analyzed and only the first 35 videos for each search keyword were evaluated. Another limitation was the daily changing statistic data on the YouTube platform. Moreover, the analyzed videos were not checked for whether the adverts or promotional products influenced the statistical parameters. Further studies could verify these problems.

## 5. Conclusions

In conclusion, it can be stated that YouTube can be a valuable source of medical information for patients and caregivers. The quality of videos mostly depends on the authorship and the source of video providers—physicians or academic and health care-related organizations provide the best quality content based on professional medical knowledge. The awareness of frailty syndrome as a real health problem, particularly among the elderly, should increase and the popularity of YouTube-based resources could help to achieve this aim.

## Figures and Tables

**Table 1:** DISCERN scoring system.

DISCERN	
Section 1	Is the publication reliable?
1	Are the aims clear?
2	Does it achieve its aims?
3	Is it relevant?
4	Is it clear what sources of information were used to compile the publication (other than the author or producer)?
5	Is it clear when the information used or reported in the publication was produced?
6	Is it balanced and unbiased?
7	Does it provide details of additional sources of support and information?
8	Does it refer to areas of uncertainty?
Section 2	How good is the quality of information?
9	Does it describe how each treatment works?
10	Does it describe the benefits of each treatment?
11	Does it describe the risks of each treatment?
12	Does it describe what would happen if no treatment were used?
13	Does it describe how the treatment choices affect overall quality of life?
14	Is it clear that there may be more than one possible treatment choice?
15	Does it provide support for shared decision-making?
16	Based on the answers to all of the above questions, rate the overall quality of the publication as a source of information about treatment choices

**Table 2:** JAMA scoring system.

JAMA Scoring System	
Authorship	Authors and contributors, their affiliations, and relevant credentials should be provided
Attribution	References and sources for all content should be listed clearly, and all relevant copyright information should be noted
Disclosure	Website “ownership” should be prominently and fully disclosed, as should any sponsorship, advertising, underwriting, commercial funding arrangements or support, or potential conflicts of interest
Currency	Dates when the content was posted and updated should be posted

**Table 3:** The GQS scores according to the source of the video uploaders.

	Low Quality	Intermediate Quality	High Quality	Total
Educational channel	2 (7%)	24 (86%)	2 (7%)	28 (38%)
Health channel	6 (24%)	11 (44%)	8 (32%)	25 (33%)
Physician channel	0	5 (50%)	5 (50%)	10 (13%)
Hospital channel	2 (22%)	6 (67%)	1 (11%)	9 (12%)
News channel	0	2 (67%)	1 (33%)	3 (4%)
TOTAL	10 (13%)	48 (64%)	17 (23%)	75

**Table 4:** The GQS groups compared to other parameters.

Quality	Duration (Seconds)	Views	DISCERN Score
low	285 (63–1168)	946 (60–2336)	2.77
intermediate	358 (42–1632)	1097 (2–11479)	3.31
high	478 (140–1087)	1261 (16–6222)	3.91

**Table 5:** The characteristics of videos according to source of uploading groups.

Source of Upload	DISCERN Mean ± SD	GQS Mean ± SD	JAMA Mean ± SD	Duration	Views	Likes	Dislikes	Comments
Physician	3.75 ± 0.39	3.75 ± 0.68	2.65 ± 1.00	509 (171–977)	1283 (40–6222)	10.60 (0–45)	0.30 (0–3)	0.70 (0–4)
News	3.69 ± 0.38	3.33 ± 1.04	2.67 ± 0.76	511 (55–1338)	63 (16–137)	0.33 (0–1)	0	0
Educational channel	3.33 ± 0.49	3.04 ± 0.58	2.73 ± 0.80	390 (42–1632)	1200 (2–6802)	5.43 (0–17)	0.21 (0–2)	0.53 (0–7)
Health channel	3.28 ± 0.52	3.06 ± 0.83	2.58 ± 0.88	328 (63–1022)	1036 (6–11479)	6.12 (0–61)	0.24 (0–2)	0.28 (0–2)
Hospital channel	3.24 ± 0.50	2.72 ± 0.62	2.78 ± 0.97	230 (65–872)	1112 (104–4419)	4.89 (0–12)	0.11 (0–1)	0.22 (0–1)

## Data Availability

The data were accessed from available for public, open-access websites.

## Appendix A

**Table A1:** The links of high-quality videos.

The Links of High-Quality Videos	
1	https://www.youtube.com/watch?v=h5WzpDfH5K8
2	https://www.youtube.com/watch?v=yIzp4pStILU
3	https://www.youtube.com/watch?v=UsFUrENDO_U
4	https://www.youtube.com/watch?v=nlAiHoXAL8c
5	https://www.youtube.com/watch?v=YclVYGMMUuA
6	https://www.youtube.com/watch?v=ux2da3ojQsA
7	https://www.youtube.com/watch?v=7ba9_QRP1Hc
8	https://www.youtube.com/watch?v=zw76IFCPYyU
9	https://www.youtube.com/watch?v=v2dgRWU9kL0
10	https://www.youtube.com/watch?v=pUmd1qAd0vw
11	https://www.youtube.com/watch?v=1-g6toBjXlI
12	https://www.youtube.com/watch?v=Ju_1QmKfz1A
13	https://www.youtube.com/watch?v=muZPodZZWtw
14	https://www.youtube.com/watch?v=3fjmd849DR0
15	https://www.youtube.com/watch?v=OySZcWmdEMM
16	https://www.youtube.com/watch?v=41cMkvsaOOM
17	https://www.youtube.com/watch?v=Cig3Oqs4TAk
